# Editorial: Cardiovascular complications of emerging oncological therapies

**DOI:** 10.3389/fcvm.2024.1530705

**Published:** 2024-12-16

**Authors:** Isaac Ho, Yee-Tat Lee, Tsun-Ho Lam, Chun-Ka Wong

**Affiliations:** ^1^ Department of Clinical Oncology, Queen Mary Hospital, Hong Kong, Hong Kong SAR, China; ^2^ Department of Medicine, School of Clinical Medicine, Cardiology Division, Li Ka Shing Faculty of Medicine, The University of Hong Kong, Hong Kong, Hong Kong SAR, China

**Keywords:** immune checkpoint inhibitor, myocarditis, hypertension, cardiovascular adverse event, cardio-oncology

**Editorial on the Research Topic**
Cardiovascular complications of emerging oncological therapies

Oncological therapies have advanced rapidly, significantly improving cancer patient survival. One of the key breakthroughs in oncology is immune checkpoint inhibitors (ICIs). By blocking interactions between programmed death-ligand 1 (PD-L1) and programmed cell death protein 1 (PD-1) or targeting cytotoxic T-lymphocyte-associated protein 4 (CTLA-4), ICIs enhance the immune system's ability to identify and eliminate cancer cells. ICIs have proven effective in treating an ever-increasing range of otherwise incurable malignancies. The overall survival benefit of immunotherapy over conventional therapies has been demonstrated in multiple large-scale phase III clinical trials, particularly in malignancies such as melanoma and PD-L1-positive lung cancer. Despite being an effective cancer treatment, ICI-related toxicities are not uncommon. One rare but potentially lethal side effect is ICI-induced myocarditis, which may occur in isolation or alongside other immune-related adverse events (IRAEs), such as the “3M” syndrome—myocarditis, myositis, and myasthenia (1). Some combinations of immunotherapies and specific dosing intervals carry a higher risk of ICI-myocarditis than others (2). While clinical characteristics and treatment responses to ICI-induced myocarditis are becoming clearer, the precise pathogenic mechanism remains elusive despite previous patient case-control studies and animal models (3, 4).

In a recently published study, Quagliariello et al. used an *in vitro* model involving co-culturing of cardiomyocytes and peripheral blood mononuclear cells (PBMC), to investigate the inflammatory response to ICI monotherapy and combinational therapies, consisting of PD-L1, PD-1, CTLA-4, and Lymphocyte-activation gene 3 (LAG-3) inhibitors. It was found that co-culture systems that were treated with combinational ICI therapy resulted in higher level of cardiac biomarkers, including troponin and brain natriuretic peptide, than those treated with monotherapy. They found that co-culture systems treated with combination ICI therapy resulted in higher levels of cardiac biomarkers, including troponin and brain natriuretic peptide, than those treated with monotherapy. Further evaluation to investigate potential mechanisms demonstrated heightened inflammatory responses with increased expression of inflammasome and cytokines. The authors also performed additional evaluation to delineate changes in mitochondrial and epigenetic domains. Altogether, the report by Quagliariello et al. provided novel insights into the potential pathogenic mechanism of ICI-induced myocarditis.

In addition to potentially causing myocarditis, ICI therapies have also been linked to other cardiovascular adverse events (CVAE). Overall, 0.80%–1.07% patients treated with ICI therapy experience CVAE (5), which potentially include myocardial infarction, stroke, arrhythmia, heart failure, myocarditis, pericardial disease, vasculitis, and hyperlipidemia (6, 7). Currently, real-world data related to ICI-associated CVAE is still scarce, and it is unclear which groups of patients are more susceptible to developing CVAE. To investigate the potential cardiotoxicities combination therapies, Inno et al. performed a meta-analysis involving 12 trials and 8,124 patients, assessing the cardiovascular toxicity of ICI plus angiogenesis inhibitors (AIs) compared to AIs alone Inno et al. It was found that patients with additional treatment with ICI had higher risk of developing severe hypertension. Reassuringly, the other analyzed CVAE, including stroke, myocardial infarction and pulmonary embolisms, were not significantly increased. Furthermore, patients treated with combination ICI and AI had superior 1-year progression free survival.

The expanding use of ICIs necessitates better understanding of their cardiovascular risks. As research priorities continue to evolve, emphasis must be placed on large-scale prospective studies to identify risk factors and biomarkers, mechanistic investigations to elucidate pathogenic pathways, clinical trials evaluating preventive and therapeutic interventions, and development of standardized monitoring protocols. As our understanding evolves, the goal remains to maximize the therapeutic benefits of ICIs while effectively managing their cardiovascular complications. This requires continued collaboration between oncologists, cardiologists, and basic scientists in the emerging field of cardio-oncology.
